# Supplementary material for: Beyond Pattern Recognition: TLR2 Promotes Chemotaxis, Cell Adhesion, and Migration in THP-1 Cells
Source: Cells. 2023 May 19;12(10):1425. doi: 10.3390/cells12101425 (PMC10217463; doi:10.3390/cells12101425)
Supplement: Supplementary file 1 [file cells-12-01425-s001.zip › Figure S2.pdf]

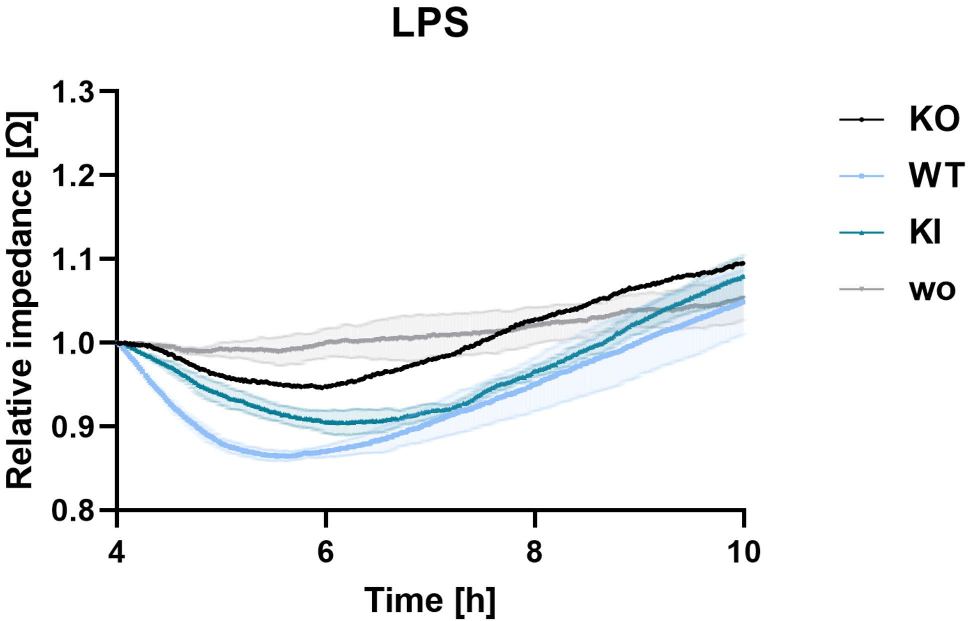

Figure S2. Endothelial barrier disruption and transendothelial migration using THP-1 WT, KO, and KI cells stimulated with LPS (100 ng/mL).
