# Supplementary figures and images for: Beyond Pattern Recognition: TLR2 Promotes Chemotaxis, Cell Adhesion, and Migration in THP-1 Cells
Source: Cells. 2023 May 19;12(10):1425. doi: 10.3390/cells12101425 (PMC10217463; doi:10.3390/cells12101425)

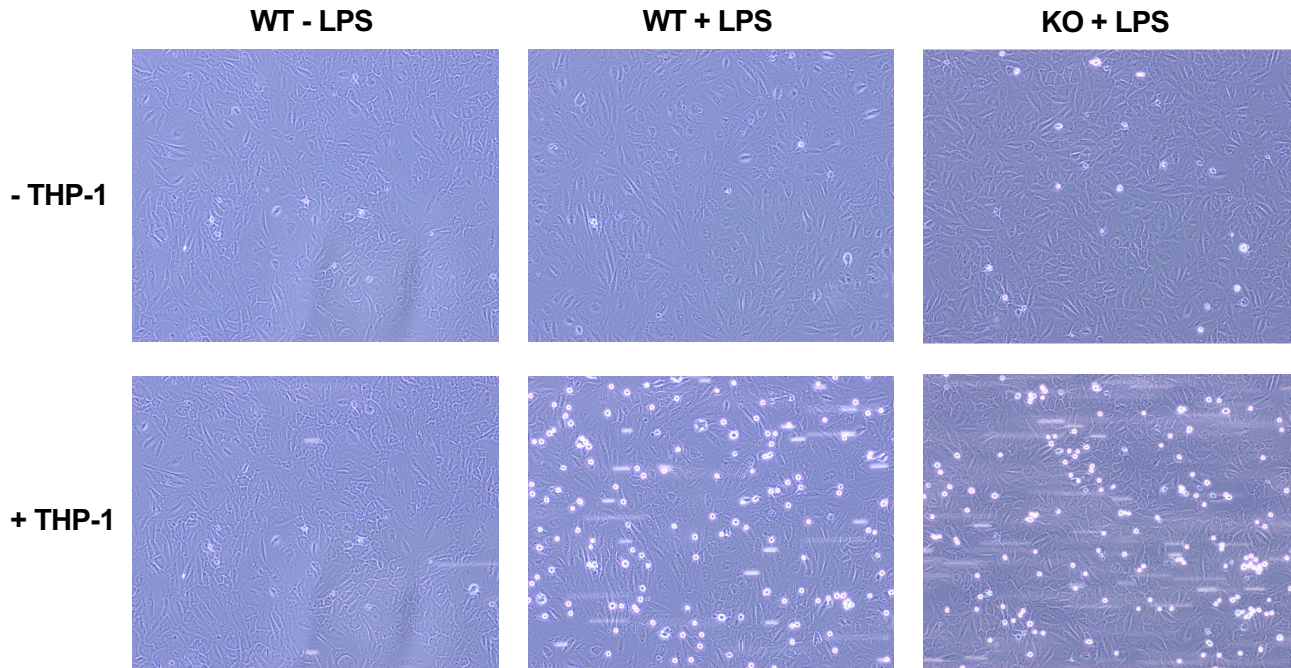

**Figure S1.** Cell layer before and after recirculation of THP-1 WT or KO cells for 15min

Supplement: Supplementary file 1 [file cells-12-01425-s001.zip › Figure S1.pdf]
